# Metal-Induced Trap States: The Roles of Interface and Border Traps in HfO_2_/InGaAs

**DOI:** 10.3390/mi14081606

**Published:** 2023-08-15

**Authors:** Huy-Binh Do, Quang-Ho Luc, Phuong V. Pham, Anh-Vu Phan-Gia, Thanh-Son Nguyen, Hoang-Minh Le, Maria Merlyne De Souza

**Affiliations:** 1 Faculty of Applied Science, Ho Chi Minh City University of Technology and Education, 01 Vo Van Ngan Street, Ho Chi Minh City 700000, Vietnam; 2 Department of Materials Science and Engineering, National Yang Ming Chiao Tung University, 1001, Daxue Road, East District, Hsinchu 300093, Taiwan; 3 Department of Physics, National Sun Yat-sen University, Kaohsiung 80424, Taiwan; 4 Faculty of Fundamental Sciences, University of Architecture Ho Chi Minh City, 196 Pasteur St., Dist. 3, Ho Chi Minh City 700000, Vietnam; 5 Faculty of electrical and electronics engineering, Ho Chi Minh City University of Technology and Education, 01 Vo Van Ngan Street, Ho Chi Minh City 700000, Vietnam; 6 EEE Department, University of Sheffield, Sheffield S10 2TN, UK

**Keywords:** defects in HfO_2_, interface traps, border traps, acceptor-like and donor-like traps, Hf 4f peaks, valence band maximum, electronic band structure

## Abstract

By combining capacitance–voltage measurements, TCAD simulations, and X-ray photoelectron spectroscopy, the impact of the work function of the gate metals Ti, Mo, Pd, and Ni on the defects in bulk HfO_2_ and at the HfO_2_/InGaAs interfaces are studied. The oxidation at Ti/HfO_2_ is found to create the highest density of interface and border traps, while a stable interface at the Mo/HfO_2_ interface leads to the smallest density of traps in our sample. The extracted values of D_it_ of 1.27 × 10^11^ eV^−1^cm^−2^ for acceptor-like traps and 3.81 × 10^11^ eV^−1^cm^−2^ for donor-like traps are the lowest reported to date. The density and lifetimes of border traps in HfO_2_ are examined using the Heiman function and strongly affect the hysteresis of capacitance–voltage curves. The results help systematically guide the choice of gate metal for InGaAs.

## 1. Introduction

Hf-based high-k oxides (Hf-based ferroelectrics) have been identified as one of the most promising candidates in future microelectronic applications [1,2,3,4,5,6,7] due to their excellent compatibility with existing Complementary Metal Oxide Semiconductor (CMOS) processes [8,9,10,11,12,13]. The functional properties of HfO_2_ depend critically on its defects: oxygen vacancies [14] or the metal/HfO_2_ [15] and HfO_2_/substrate [3] interfaces. A low density of oxygen vacancies is required as dielectric for low power-consumption devices [2,3], whereas high density is attractive in ferroelectric FETs and resistive random access memory (ReRAM) [16,17,18]. The conductivity of HfO_2_-based materials is believed to be governed by oxygen vacancies via the formation of a conducting filament [19,20]. A change in local band structure [21], oxygen vacancy-induced trap states [22], conducting oxide [23], and crystalline suboxide phases [24] all contribute to explaining the conductivity of HfO_2_-based materials.

Among III-V materials, InGaAs is a promising epitaxial layer due to its high electron mobility and small band gap of 0.74 eV [25] in high-speed and low-power logic technologies [26,27,28,29]. InGaAs quantum well metal oxide semiconductor field effect transistors (MOSFETs) with a gate length of 70 nm were shown to yield a current gain of ~1 × 10^4^ by J. Lin et al. [26]. X. Cai et al. reported that a high mobility of 570 cm^2^/V·s can be reached in InGaAs FinFETs at a fin width of 7 nm. Gate oxide trapping was believed to degrade the device’s performance [27]. In logic applications, InGaAs gate-all-around MOSFETs with sub-10 nm top-width nanowires were investigated with the subthreshold voltage of 70 mV/dec [28]. Unlike a favorable Si/SiO_2_ interface in silicon, there is a lack of native oxide in InGaAs, leading to techniques in plasma treatment of the surface [2], passivation using sub-nanometer AlN [30], and chemical treatment [31] to achieve interface engineering. This approach is based on the effect of the InGaAs epitaxial layer on the properties of HfO_2_ layers. However, gate metals may also induce trap states at the metal/HfO_2_ interface [15], facilitating the diffusion of the oxygen vacancy in HfO_2_ films. The passivation of the metal/HfO_2_ interface was conducted using the AlN layer [4], resulting in an increase in the permittivity of HfO_2_ of 47%. Studies of bias temperature instability revealed the role of deep states in high-k to degradation [32]. Although there are many studies of interface traps at HfO_2_/InGaAs arising from the interaction between HfO_2_ and the InGaAs layer, the behavior of interface traps and border traps due to the effects of gate metals have not been systematically examined. Studying these effects is required not only in logic but also resistive random-access memory (RRAM) devices.

To answer the questions raised by a metal-induced interface and border traps in HfO_2_ and at the HfO_2_/InGaAs interfaces, the electrical properties of HfO_2_ in metal/HfO_2_/InGaAs structure are investigated in this study using metal oxide semiconductor capacitors (MOSCAPs). A TCAD simulation is adapted to simulate electrical characteristics and the band structure of devices. The properties of interface traps and border traps are extracted from a comparison of the theoretical capacitance–voltage (CV) characteristics and experiments. The material properties of HfO_2_ are investigated using transmission electron microscopy (TEM) and X-ray photoelectron spectroscopy (XPS) spectra. Based on material and electrical investigations, we explain systematically the role of gate metals in the formation of acceptor-like and donor-like traps at the HfO_2_/InGaAs interface, as well as the border traps within HfO_2_.

## 2. Experimental Procedure and TCAD Simulation

### 2.1. Experimental Procedure

**Figure 1:**
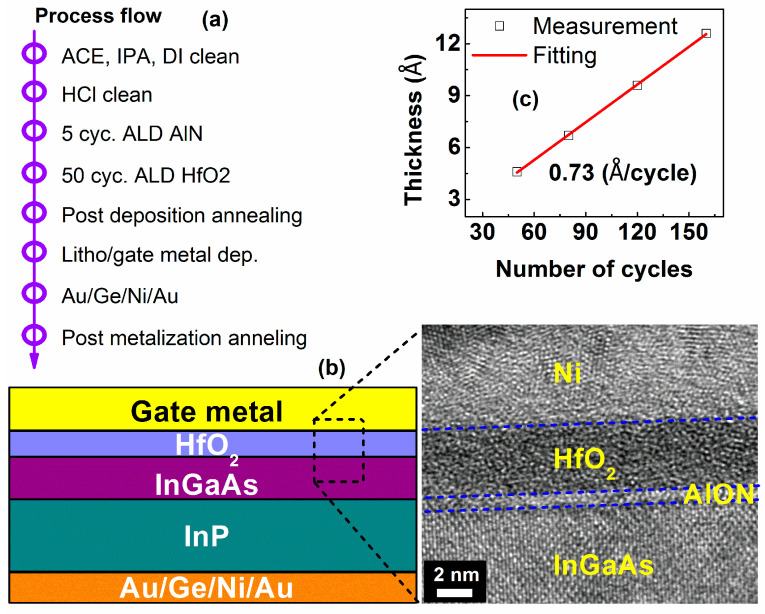
(**a**) Process of fabrication of HfO_2_/In_0_._53_Ga_0_._47_As MOSCAPs. (**b**) The schematic cross-section and HRTEM image of the metal/HfO_2_/In_0_._53_Ga_0_._47_As structure after PDA. (**c**) The relationship between HfO_2_ thickness and the number of cycles in the ALD chamber. The growth rate was determined at ~0.73 Å/cycle.

In_0_._53_Ga_0_._47_As (IGA) MOSCAPs were fabricated following the process flow shown in Figure 1a. The channel layer is a commercial 100 nm n-doped In_0_._53_Ga_0_._47_As (5.0 × 10^17^ cm^−3^ Si-doped) layer grown on n+-InP substrate by solid source molecular beam epitaxy. Acetone (ACE) and isopropanol (IPA) were used to clean the IGA surface before the native oxide was etched in dilute HCl. The samples were subsequently loaded into an ALD chamber for the deposition of high-k with a growth temperature of 250 °C. The deposition process includes 2 steps: (i) 5 cycles of the AlN layer with a thickness of ~0.7 nm, as shown in Figure 1b, and (ii) HfO_2_ layers, in which the thicknesses varied from 50 cycles to 150 cycles. The growth rate of HfO_2_ was determined to be ~0.73 Å/cycle, as shown in Figure 1c. All of the thicknesses of HfO_2_ were measured and fitted using a SOPRA GES5 Ellipsometer because of the large number of samples. Thicknesses of 50 cycles of HfO_2_ samples are double checked using the HRTEM. Post-deposition anneal (PDA) was conducted at 450 °C for 2 min in forming gas (FG) after the high-k deposition. The HRTEM in Figure 1b indicates that the atomically sharp and clear Ni/HfO_2_/AlON/In_0_._53_Ga_0_._47_As interface of the PDA sample was obtained with virtually no interdiffusion. HfO_2_ film was found to be amorphous in Figure 1b, which is in agreement with the report [33]. Finally, a contact was defined on HfO_2_ via lithography/lift-off processes, followed by a post-metallization anneal at 250 °C for 30 s in FG.

### 2.2. Simulation of CV Characteristics

HfO_2_/IGA MOSCAPs were simulated using the Silvaco TCAD 2-D device simulation tool. The flowchart of the process simulation is shown in Figure S1 in the supporting information. The structure of the MOSCAPs is illustrated in Figure 1b. To consider the effects of the high field on mobility and carrier concentration on carrier lifetime, a high field model and a concentration-dependent lifetime model were adopted, respectively. The Auger recombination model was utilized to account for the effect of high electron concentration (N_e_). The Shockley–Read–Hall model was used to simulate the trap-assisted recombination. The band gap narrowing effect was included in the simulation to consider a shrinkage of the bandgap occurring when the impurity concentration is particularly high. To make sure that the models being used represent and replicate a real-world device accurately, TCAD parameters (shown in Table 1) were calibrated against the device structure in Figure 1b through capacitance–voltage (CV) characteristics. The electron affinity of HfO_2_ was used based on the extracted valence band offset of the HfO_2_/IGA heterostructure from XPS measurements, and the permittivity of HfO_2_, corresponding to different gate metals, were extracted from the curve of measured capacitance-equivalent thickness (CET) versus HfO_2_ thickness. The parameters of IGA are default values from Silvaco TCAD.

## 3. Results and Discussion

### 3.1. Experimental Electrical Behavior

**Figure 2:**
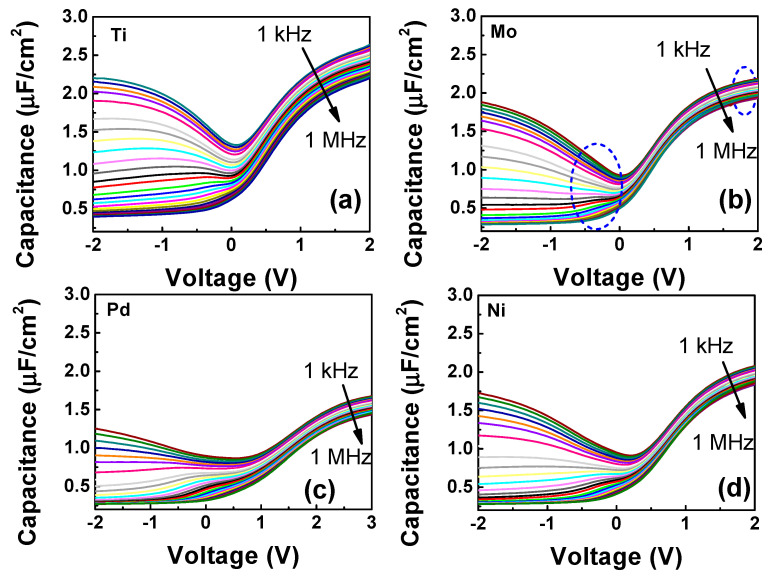
Multi-frequency (1 kHz–1 MHz) C-V characteristics of MOSCAPs with different gate metals: (**a**) Ti, (**b**) Mo, (**c**) Pd, and (**d**) Ni. A hump around 0 V and high C-V dispersion is found in the Ti sample, indicating the presence of high densities of interface (D_it_) and border traps (N_BT_). Oppositely, the lowest capacitance is in the Pd sample, referring to the degradation of permittivity of HfO_2_ when Pd is used as a gate metal. The best C-V behavior without hump around 0 V and small C-V dispersion is in the Mo sample (highlighted by blue circles), implying the lowest D_it_ and N_BT_.

Figure 2 shows multi-frequency (1 kHz–1 MHz) C-V characteristics with different gate metals whose work functions are 4.33 eV (Ti), 4.95 eV (Mo), 5.10 eV (Pd), and 5.15 eV (Ni) [36]. It is seen that except for sample A, all other samples provide quite similar C-V characteristics. The high-frequency dispersion in the accumulation of sample A is due to high densities of border traps in the bulk of the dielectric [37,38]. Samples A, C, and D exhibit typical ‘humps’ around 0 V of some C-V curves, indicating the presence of interface traps, which can be suppressed effectively by increasing the frequency [39]. The minimal frequency dispersion of the accumulation capacitance in sample D and the smallest hump around 0 V in sample B qualitatively indicate that Ni and Pd gate metals induce small densities of interface (D_it_) and border traps (N_BT_). The effects of gate metals on the interface and border traps are going to be analyzed in the simulation section. At 1 kHz, a high inversion capacitance equal to the oxide capacitance (C_ox_) in sample B indicates that minority carriers respond freely to the signal to form a fully inverted layer in agreement with previous reports [40].

**Figure 3:**
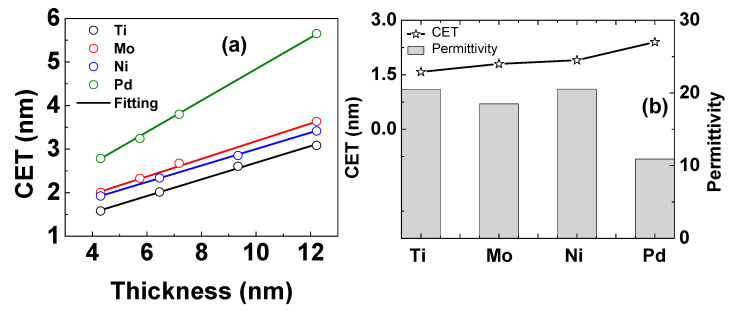
(**a**) Equivalent capacitance thickness (CET) versus physical oxide thickness of all samples. (**b**) Equivalent capacitance thickness (CET) and permittivity of all samples for a physical oxide thickness of 4.3 nm.

Figure 3a shows the capacitance oxide thickness (CET) at +2 V and 1 MHz versus the HfO_2_ thickness (*t_HfO_*_2_) curves of all samples. The total thickness of HfO_2_ and AlON in this study is large enough to neglect the quantum effect [41]. The charge centroid is expected to be at the AlON/InGaAs interface when identifying the thickness of the oxide in accumulation. CET is a function of the HfO_2_ thickness (*t_HfO_2__*) following Equation (1).

(1) $$\;CET=\frac{\varepsilon_{SiO_{2}}}{\varepsilon_{HfO_{2}}}t_{HfO_{2}}+\frac{\varepsilon_{SiO_{2}}}{\varepsilon_{AlON}}t_{AlON}+\varepsilon_{ox}\frac{\partial \left(\Psi_{s}-\Psi_{s,G}\right)}{\partial \left(D_{s}\right)}$$

where $\varepsilon_{HfO_{2}}$ and *t_AlON_* are the permittivity of the HfO_2_ and the thickness of *AlON*, respectively, $\varepsilon_{SiO_{2}}=3.9$, Ψ*_S_* and Ψ*_S,G_* are the band bending at the channel and gate electrodes, respectively, and *D_S_* is the electric displacement in the substrate just beneath the oxide/InGaAs interface. Equation (1) allows the permittivity of the high-k layer to be extracted based on the slopes of the CET-t_ox_ curves in Figure 3a, without knowing the band bending at the channel and gate electrodes. The extracted values of permittivity are shown in Figure 3b. The values of permittivity of the Ti-, Mo-, and Ni-gated samples are the same, fluctuating around 18.5 to 21, which is in agreement with a previous report [42]. The permittivity of the Pd sample is ~11, too small to compare to others, which is likely due to the formation of a PdO layer [15] with a small permittivity of ~8 [43].

### 3.2. Effects of Gate Metals on Interface Traps and Border Traps

The impact of fast and slow traps on the electrical properties of HfO_2_ on IGA is examined via TCAD simulations using the parameters shown in Table 1. Interface and border traps are included at the AlON/IGA interface and within the band gap of *HfO*_2_ (for example, oxygen vacancies), respectively, to fit the simulation C-V curves to the ones observed in the experiment. The effects of the AlON/HfO_2_ interface traps on C-V curves are not separately investigated in this study because it is hard to distinguish this type of trap from others. Instead, this trap and border trap are considered as a single object. Additionally, a negative interface fixed charge of around 5.0 × 10^11^ cm^−2^ to 1.5 × 10^12^ cm^−2^, similar to that in a recent study, is included [44]. The interface fixed charges are related to the incorporation of nitrogen in high-k dielectrics during ALD deposition or in post-deposition treatment [45,46]. This charge is treated as a sheet of charge at the interface controlled by the interface boundary condition, which causes the shift of the C-V curves. On the other hand, interface traps and bulk traps are added as space charge directly into the right-hand side of Poisson’s equation:

(2) $$div\left(\varepsilon \nabla \Psi \right)=-\rho =q\left(n-p-N_{D}^{+}+N_{A}^{-}\right)-Q_{T}$$

where Ψ is the electrostatic potential, *ε* is the permittivity, *ρ* is the local space charge density, $N_{D}^{+}$ and $N_{A}^{-}$ are the ionized donor and acceptor impurity concentrations, respectively, and *Q_T_* is the charge due to traps and defects. The total charge caused by the presence of traps in the right-hand side of Poisson’s equation can be defined by:

(3) $$Q_{T}=q\left(N_{tD}^{+}-N_{tA}^{-}\right)$$

where $N_{tD}^{+}$ and $N_{tA}^{-}$ are the densities of the ionized donor-like and acceptor-like traps, respectively.

**Figure 4:**
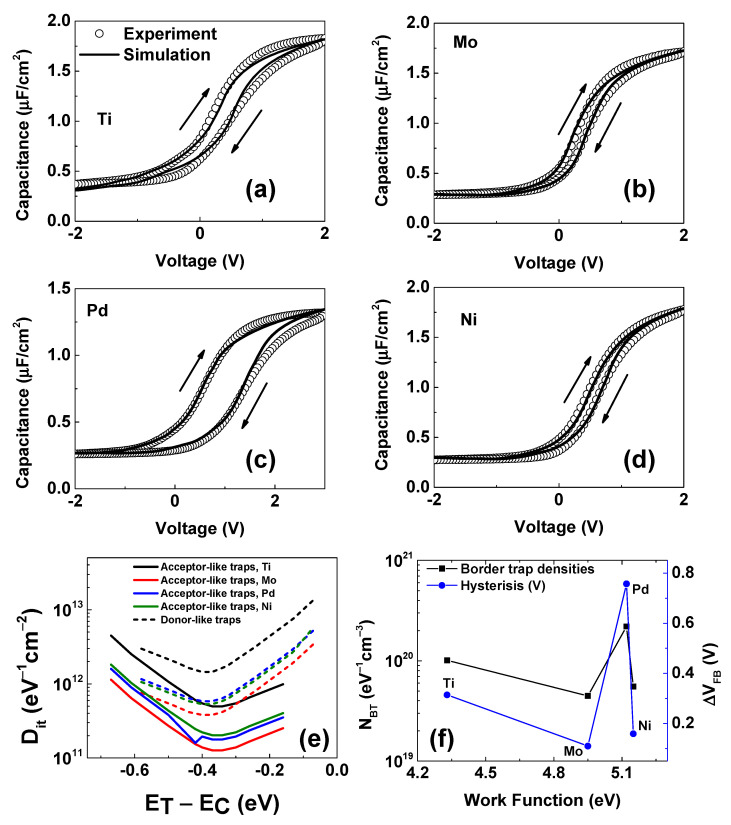
Results of C-V characteristics at 1 MHz from TCAD simulations and experiment of (**a**) Ti, (**b**) Mo (**c**) Pd, and (**d**) Ni samples. The up and down arrows illustrate the forward and reverse sweep of C-V curves, respectively. (**e**) Densities of interface traps extracted from TCAD simulations of the C-V curves in (**a**–**d**). (**f**) The densities of border traps and the difference in flat band voltage of the samples are shown in (**a**–**d**).

It is seen in Figure 4a–d that the simulation results fit well with the experiment. The process to calibrate the simulation curves consists of two steps: (i) adding interface traps to the AlON/IGA interface to fit C-V curves in the forward bias and (ii) simulating C-V curves in reverse bias to obtain hysteresis by adding border traps in the bandgap of HfO_2_. There are two types of interface traps: donor-like and acceptor-like. A donor-like trap can be either positive or neutral, and it is positively charged (ionized) as empty and neutral as filled (with an electron). An acceptor-like trap can be either negative or neutral, and it is neutral as empty and negatively charged (ionized) as filled (with an electron). One of the well-known methods to extract densities of interface traps (D_it_) is the conductance method. This method is based on estimating the peak of equivalent parallel conductances calculated from measured impedance [47]. The advantage of this method is that the D_it_ can be extracted directly from experimental data. However, the extracted D_it_ is reported to be not correct in the case of large D_it_, especially when C_ox_ < qD_it_ [47]. Another popular method is the Terman method. To extract D_it_, the ideal C-V curve is plotted. The stretch-out of the experimental curve as compared to the ideal one provides the value of D_it_. The D_it_ extracted by the Terman method is typically larger (~10 times) than that extracted by the conductance method [4].

Figure 4e shows the D_it_ of all samples extracted from TCAD simulations. It is seen that D_it_ is minimal at the mid gap, and acceptor-like densities are smaller as compared to donor-like densities for all samples. The lifetime of interface traps is extracted to be 2.9 × 10^−9^ s, which is the same as the previous study [48]. The smallest D_it_ (1.39 × 10^11^ eV^−1^cm^−2^ acceptor-like traps, and 3.81 × 10^11^ eV^−1^cm^−2^ donor-like traps) is in the sample with a Mo gate. This result is in accordance with the multi-frequency C-V curves in Figure 2b, where the Mo sample seems to not present any hump around 0 V at high frequency. In contrast, the highest D_it_ is in the Ti-gated sample, corresponding to the largest hump around 0 V at a high frequency, as shown in Figure 2a. The fact that the D_it_ from donor-like traps near the valence band edge of all samples is much smaller compared to those near the conduction band edge confirms the strong inversion of the multi-frequency C-V curves of all samples shown in Figure 2, implying freely moving minority carriers in the channel. In our previous report, a combination of Mo/Ti gate metal and the passivation layer AlN between Ti and HfO_2_ leads to a small D_it_ [4]. In that study, a thin AlN layer was believed to prevent the reaction between Ti and HfO_2_, diminishing the formation of oxygen vacancies in HfO_2_.

The extracted border traps are shown In Figure 4f. Because the border traps near the conduction band strongly affect the n-type substrate, only acceptor-like border traps are considered in this study [49]. To simulate the hysteresis of C-V curves, the Heiman method [50] is utilized. The traps are assumed to have a uniform distribution with depth in HfO_2_, but the capture cross-section $\sigma_{nx}$ and the lifetime of traps $\tau_{T}$ for electrons are a function of distance from the AlON/IGA interface:

(4) $$\sigma_{nx}=\sigma_{n}e^{-2k_{e}x}$$

(5) $$\tau_{T}=\frac{1}{n_{s}\bar{v}\sigma_{nx}}$$

where *x* is the distance from the interface to the trap position in HfO_2_, $\sigma_{n}$ is the capture cross-section of traps for electrons at the interface, *n_s_* is the trap concentration at the interface, $\bar{v}$ is the thermal velocity of electrons, and $k_{e}$ is determined from:

(6) $$k_{e}^{2}=\frac{2m_{e}^{*}\left(E_{C}-E_{tA}\right)}{\hbar^{2}}$$

where *E_C_*, m*_e_, *E_tA_*, ℏ, and *E_tA_* are the conduction band minimum, the effective mass of the electron, the border trap level, the Planck constant, and the level of the acceptor-like trap, respectively. It is seen that the largest border traps of 2.2 × 10^20^ eV^−1^cm^−3^ occur in the Pd sample, explaining the largest hysteresis of 0.76 V of CV curves in Figure 4c. The extracted lifetime of border traps at the interface is 2.5 × 10^−5^ s, which is larger than the interface traps. The value of lifetime is the same as in the study of Zhang et al. [51]. For the border traps in high-k, the values of lifetimes are calculated in Equation (5). Similar to the extracted D_it_, the smallest value of border traps, an N_BT_ of 4.46 × 10^19^ eV^−1^cm^−3^, also occurs in the Mo sample, which is confirmed by the smallest hysteresis in its C-V hysteresis of 0.11 V, as shown in Figure 4f. Although the work function of Ni and Pd is nearly the same, the quality of the Ni sample is much better than Pd. The reasons for the degradation have been discussed in our previous report, where the formation energy of the metal oxygen defect has a critical role that stabilizes the metal/high-k interface [15].

**Figure 5:**
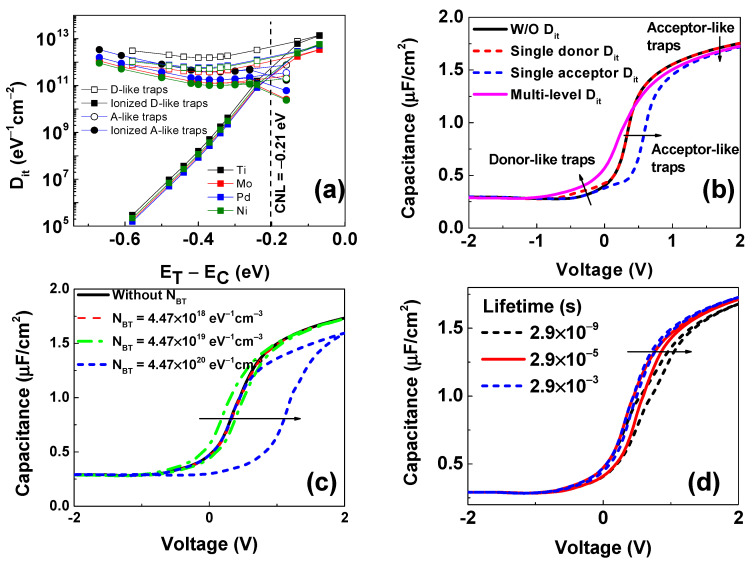
(**a**) The distribution of ionized donor-like and acceptor-like traps at the AlON/InGaAs interface. Trap positions are compared with the conduction band minimum of InGaAs. (**b**) Effects of the kinds of interface traps on the C-V behaviors. Impacts of (**c**) densities and (**d**) lifetime of border traps on the C-V behaviors. The black arrows highlight the shift of C-V curves under the effects of interface traps, border traps, and the lifetime of traps.

**Figure 6:**
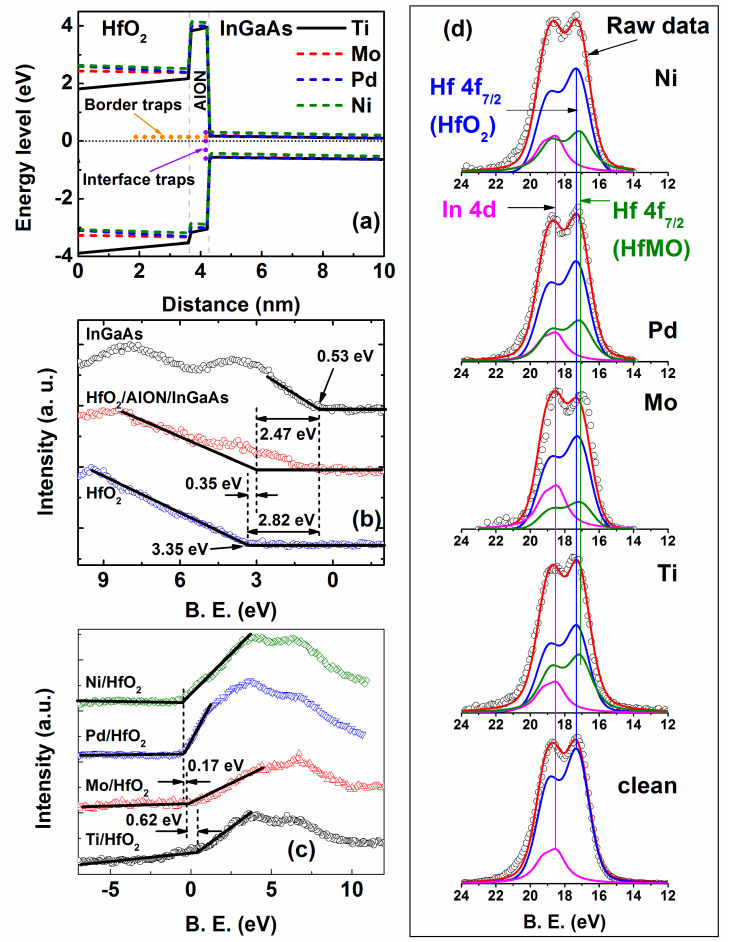
(**a**) Band diagram of a metal/HfO_2_/AlON/InGaAs structure extracted by TCAD simulation. (**b**) Valence band maxima of an HfO_2_/AlON/IGA structure derived from XPS measurement. (**c**) Valence band maxima of metal/HfO_2_ structures derived from XPS measurement. (**d**) Hf 4f_7/2_ of clean HfO_2_ surface and (Ti, Mo, Pd, Ni)/HfO_2_ interfaces.

The effects of D_it_ and N_BT_ are systematically studied in Figure 5. Figure 5a indicates that the ionization profiles of interface traps extracted from the TCAD simulation depend on the types of traps and their positions as compared to the charge neutrality level (CNL) at 0.21 eV inside the IGA band gap [52]. It is seen that donor-like traps are ionized above CNL, creating a positive charge at the interface. A complete ionization of the interface of donor-like traps generates a net positive charge, contributing to the total capacitance, which induces a negative shift and up-shift of C-V curves (pink solid line) compared to the ideal (black solid line) at a negative voltage, as shown in Figure 5b. Based on the stretch-out of C-V curves due to these shifts, it was reported that densities of interface traps could be extracted by the Terman method [47]. However, in our case, it was impossible to separate the densities of ionized and neutral traps using this method. Opposite to donor-like traps, acceptor-like traps are filled below the CNL, as shown in Figure 5a. These traps induce a net negative charge at the interface, the same as the interface-fixed charge, when they are completely ionized. These traps also cause a right shift of the C-V curve and a decrease in the accumulation capacitance, as shown in Figure 5b (pink solid line). To distinguish the effects of donor-like and acceptor-like traps on the charge density of the channel below HfO_2_, donor-like traps (D_it_ = 3.81 × 10^11^ eV^−1^cm^−2^) or acceptor-like traps (D_it_ = 1.27 × 10^11^ eV^−1^cm^−2^) are added at the mid gap of the Mo sample in our C-V simulation. It is clear that single donor-like traps shift the C-V curves up around 0 V (red dash line). This shift means that a hump around 0 V can be created when the D_it_ of donor-like traps is high and the measured frequency is small, as seen in the experiment in Figure 2a,c,d. The blue dashed line in Figure 5b illustrates that acceptor-like traps act as a sheet of a negative fixed charge and shift the C-V curves to positive voltage. The effect of the density of border traps on the C-V curves of the Mo sample is shown in Figure 5c. In all simulations, border traps are assumed to distribute in the dielectric with a maximum depth of 2 nm from the interface, and the continuously distributed states are represented by 20 discrete trap states along a depth of 2 nm, as shown in Figure 6a. It is seen that there is no C-V hysteresis when N_BT_ = 4.47 × 10^18^ eV^−1^cm^−3^. The change in the order of N_BT_ causes a significant variation of the hysteresis, and the hysteresis larger than 1.0 V is found as N_BT_ = 4.47 × 10^20^ cm^−3^. The impact of a lifetime of border traps is considered in Figure 5d. Because only acceptor-like border traps near the conduction band edge are investigated, the lifetime affects meaningfully the accumulation region. Since the simulated frequency is 1 MHz, a lifetime of 2.9 × 10^−3^ s induces no consequence on C-V curves. The border traps have a substantial response if they have a lifetime smaller than 2.9 × 10^−5^ s, which is in agreement with the literature [51].

### 3.3. Properties of Metal/HfO_2_/AlON/In_0_._53_Ga_0_._47_As Structures

Band diagrams of all samples were extracted from the TCAD simulation at 0V, as shown in Figure 6a. The simulated band offsets of the HfO_2_/AlON/IGA structures were compared with data from XPS measurements, as shown in Figure 6b. The band offsets of the AlON/IGA and HfO_2_/AlON structures are extracted to be 2.47 eV and 0.35 eV, respectively, showing agreement between the experiment and simulation. Although five cycles of the AlN layer were deposited on IGA for passivation, XPS revealed that this layer was AlON [3]. It is found that low and high work function gate metals induce opposite bending in the band diagram. This bending depends on the flat band voltage described by [53]:

(7) $$V_{FB}=\varphi_{m,\mathrm{eff}}-\varphi_{S}-\left(Q_{f}\frac{\varepsilon_{SiO_{2}}}{\varepsilon_{ox}^{2}}t_{ox}+\frac{1}{2}\rho_{ox}\frac{\varepsilon_{SiO_{2}}}{\varepsilon_{ox}^{2}}t_{ox}^{2}\right)$$

where *V_FB_* is the flat band voltage, *ϕ*_m_ is the work function of metals, *ϕ*_S_ is the semiconductor work function, *Q_f_* is the interface fixed charges, *ε_SiO_*_2_ is the permittivity of SiO_2_, *ε_ox_* is the permittivity of HfO_2_, *t_ox_* is its thickness, and *ρ_ox_* is bulk HfO_2_ charge density. The relationship between V_FB_ and tox was reported to be linear in the HfO_2_/IGA moscap, so the effect of $\rho_{ox}$ on *V_FB_* is much smaller compared to $Q_{f}$ on *V_FB_* [3]. Because the density of interface fixed charge used to calibrate the C-V curves is quite similar ~10^12^ cm^−2^ in all samples, bending in the band diagram should be from the difference in metal work functions and the bulk charges shown in Figure 4f alone. Additionally, the border acceptor-like traps studied in this work are empty at 0 V, as shown in Figure 4f, referring to its neutral state at 0 V above the CNL. These trap charges also need a delay time to fill up. The opposite band bending (0.62 eV–0.89 eV) of the samples shown in Figure 6a is in agreement with the C-V curves in Figure 4a–d, illustrating a negative C-V shift of the Ti sample and a positive C-V shift of the others. This bend bending is also confirmed by the valence band maxima of XPS spectra measured at metal/HfO_2_ interfaces, as shown in Figure 6c.

To analyze the interaction of the metal/HfO_2_ interface, the factor that most impacts D_it_ and N_BT_ is the convolution of the Hf 4f peaks shown in Figure 6d. All samples with a diameter of ~5 mm were mounted on an XPS holder using copper tape. The pressure of the XPS chamber is ~10^−9^ torr when the measurement was conducted. For the analysis, the core levels were determined by using XPSPEAK with a Gaussian–Lorentz line shape and Shirley background, with an uncertainty of the core position of 0.05 eV. Sample charging effects were corrected by placing the C 1s peak at a binding energy (BE) of 284.8 eV and shifting the rest of the regions accordingly. The binding energy, the spin–orbit splitting (SOS), and the full-width at half-maxima (FWHM) values used to fit the curves of the two samples are shown in Table 2. For the clean HfO_2_ sample, there are two peaks that are convoluted: an Hf 4f_7/2_ peak at 17.12 eV [3,54] and an In 4d peak at 18.47 eV [3,55], representing the In-O bond. The presence of an In peak with an area of ~10% is believed to be due to In diffusion from the InGaAs layer [55]. In the other samples, Hf 4f_7/2_ peaks were split into three peaks. One of them is the Hf 4f_7/2_, which appears in the clean HfO_2_ sample, denoted as a peak of deposited HfO_2_. The In-O peak in other samples was kept to have the same BE, SOS, FWHM, and area to ensure that it does not affect other peaks in the fitting process. It is seen that a new peak at 16.97 eV dominates in all samples (besides the peak of Hf 4f_7/2_), except in clean HfO_2_. The BE of this peak is in a range of the Hf 4f_7/2_ peak (from 14.3 eV for Hf metal to 18.3 eV for perfect HfO_2_ oxide) [56], so it can be inferred that gate metals react with the oxygen in HfO_2_, producing a complex interface that contains HfMO (M: Ti, Mo, Pd, and Ni). We denote Hf 4f_7/2_ in clean HfO_2_ and HfMO as Hf1 and Hf2 peaks, respectively. The area ratio of Hf2 and Hf1 peaks, A_Hf2_/A_Hf1_, should give us information relating to the oxidation of the gate metals because more oxidation causes a higher intensity of the Hf2 peak. The area ratios A_Hf2_/A_Hf1_ in the Ti, Mo, Pd, and Ni samples were extracted at 0.775, 0.296, 0.460, and 0.390, respectively, implying that the the Mo sample created the most stable interface on HfO_2_. Its XPS result is in agreement with its C-V behavior shown in Figure 2b, with the lowest values of D_it_ and N_BT_ shown in Figure 4e,f. The stability of the Mo/HfO_2_ and Ni/HfO_2_ interfaces is also confirmed in the TEM measurement shown in Figure S2b,d (supporting information). Oppositely, the Ti/HfO_2_ interface has the highest ratio, A_Hf2_/A_Hf1_, indicating a strong reaction between Ti and O_2_^−^ in HfO_2_. This reaction is observed in Figure S2a (supporting information), showing the increase in roughness at the Ti/HfO_2_ interface. This reaction can be suppressed by passivating the HfO_2_ surface using an ultra-thin AlN layer, which was reported in our previous work [4]. The area ratio A_Hf2_/A_Hf1_ in the Pd sample is slightly higher than the Ni sample, indicating that Pd-O facilitates oxidation at the AlON/IGA interface due to the transportation of oxygen vacancies in HfO_2_. Yoshida et al. reported that Pd-O induced a thin In-O layer between HfO_2_ and IGA [57]. The effect of Pd-O on the HfO_2_ layer should be similar to this study. However, the TEM image in Figure S2c does not show the In-O layer. Instead, Pd-O causes the defects in the AlON layer, illustrated by the white arrows in Figure S2c, supporting information. The difference in the formation of the In-O layer at the high-k/IGA interface is due to the fact that AlON is an excellent passivation layer [1]. The results indicate that the reaction at the metal/HfO_2_ interface is the main factor that creates an oxygen vacancy in HfO_2_, contributing to high D_it_ at the HfO_2_/IGA interface and high N_BT_ in bulk HfO_2_. The high value of N_BT_ is a disadvantage in devices for logic applications but an advantage in memory devices.

**Figure 7:**
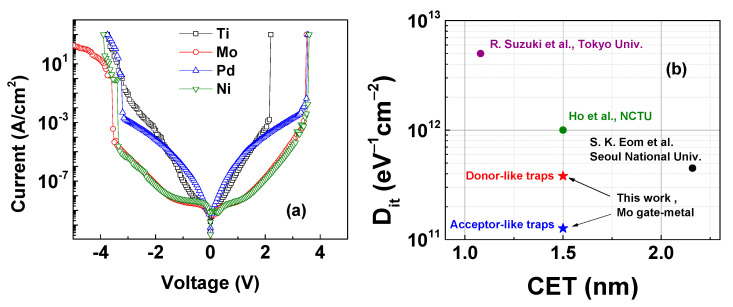
(**a**) I-V characteristics of M/HfO_2_ MOSCAPS, M = Ti, Mo, Pd, Ni. (**b**) Benchmark of D_it_ as a function of CET of Mo/HfO_2_/IGA MOSCAPs [2,59,60].

Figure 7 shows the leakage current through HfO_2_. These currents can be described by the tunneling effects in the Fowler–Nordheim regime [58]:

(8) $$J_{t}=\frac{J_{0}}{\Phi_{B}}E_{ox}^{2}exp\left(-\frac{k\Phi_{B}^{3/2}}{E_{ox}}\right)$$

where $\Phi_{B}$ and $E_{ox}$ represent the tunneling barrier and oxide electric field, respectively. It is clear that the Ti sample has high leakage current shown in Figure 7a due to the lower barrier of the AlON layer shown in Figure 6a and the high value of high D_it_ and N_BT_ shown in Figure 4e,f. Oppositely, the Mo and Ni samples have the lowest leakage current. These two samples have the same band bending shown in Figure 7a and the low D_it_ and N_BT_ shown in Figure 4e,f. For logic applications, D_it_ can be benchmarked as a function of CET, as shown in Figure 7b. Our extracted value is revealed to be the smallest (1 × 10^11^ eV^−1^cm^−2^ for acceptor-like traps) compared to other reports.

## 4. Conclusions

In summary, the impact of gate metals on interface traps and border traps in the metal/HfO_2_/AlN/InGaAs structure was systematically studied. Due to oxidation at the metal/HfO_2_ interface, low work function metal Ti creates the highest N_BT_ in HfO_2_, while the middle work function metal Mo induces the lowest D_it_ and N_BT_. The TCAD simulations confirm the occupation of traps around the CNL, where donor-like traps are ionized above CNL and acceptor-like traps are ionized below this level. The extracted lifetime of interface and border traps in this study are 2.9 × 10^−9^ s and 2.9 × 10^−5^ s, respectively. Interface traps distort the C-V characteristics, whereas border traps cause their hysteresis. The Heiman method was utilized to investigate the border traps in HfO_2_. The lowest D_it_ and N_BT_ were found to be 1.39 × 10^11^ eV^−1^cm^−2^ and 4.46 × 10^19^ eV^−1^cm^−3^, respectively, for middle work function metal Mo due to a stable interface observed in XPS as well as a flat band voltage of 0 V. The results of this study provide guidance in the choice of gate metal for microelectronic devices and random-access memory.

## Figures and Tables

**Table 1:** Parameters used in HfO_2_/IGA MOSCAPs.

Material Parameters	HfO_2_	AlON	In_0.__53_Ga_0.__47_As ^1^
Band gap (eV)	5.7 [34]	7 [34]	0.734
Electron affinity (eV)	2.52 ^2^	0.88^2^	4.67
Permittivity	10.9–21 ^3^	8.8 [34]	13.9
Effective density of state in the conduction band (cm^−3^)	-	-	2.1 × 10^17^
Effective density of state in the valence band (cm^−3^)	-	-	7.7 × 10^18^
Intrinsic carrier concentration (cm^−3^)	-	-	8.72 × 10^11^
Electron mobility (cm^2^/V·s)	-	-	10^4^
Effective electron mass	-	-	0.041 m_o_
Effective hole mass			0.46 m_o_
Tunnelling effective masses	0.17 m_o_ [35]	0.35 m_o_ [35]	-
Saturated electron drift velocity (cm/s)	-	-	2.5 × 10^7^
Saturated hole drift velocity (cm/s)	-	-	7.7 × 10^6^

^1^ Parameters of InGaAs are default values from TCAD. ^2^ Based on the band offset of HfO_2_/InGaAs, extracted from XPS spectra. ^3^ Values extracted from CET thickness curves.

**Table 2:** BE, SOS, and equal FWHM values were applied to fit the curves in Figure 6d (eV).

	Hf 4f_7/2_ (Clean HfO_2_)	In-O	Hf 4f_7/2_ (HfMO), M = Ti, Mo, Pd, Ni
BE (eV)	17.12	18.47	16.97
SOS (eV)	1.68	0.8	1.68
FWHM	1.62	1.28	1.68

## Data Availability

The data that support the findings of this study are available from the corresponding author upon reasonable request.

## Supplementary Materials

The following supporting information can be downloaded at https://www.mdpi.com/article/10.3390/mi14081606/s1, Figure S1: flow chart of process simulation in Silvaco TCAD to study the effects of interface and border traps in metal/HfO2/AlON/IGA structures.; Figure S2: TEM cross sections of metal/HfO_2_/AlON/IGA structures. Metal: (a) Ti, (b) Mo, (c) Pd, (d) Ni. The Mo/HfO_2_ and Ni/HfO_2_ interfaces were found to have no interdiffusion, while the oxidation was observed at Ti/HfO_2_ interface for Ti sample (indicated by white arrows). White arrows in Figure S2(c) demonstrate that Pd gate metal induces many defects in AlON passivation layer.
