# Supplementary material for: Metal-Induced Trap States: The Roles of Interface and Border Traps in HfO2/InGaAs
Source: Micromachines (Basel). 2023 Aug 15;14(8):1606. doi: 10.3390/mi14081606 (PMC10456933; doi:10.3390/mi14081606)
Supplement: Supplementary file 1 [file micromachines-14-01606-s001.zip › micromachines-2428332-supplementary.pdf]

Supporting information:

Metal-Induced Trap States: The Roles of Interface and Border Traps in HfO_2/InGaAs

A. Flow chart of process simulation in Silvaco TCAD

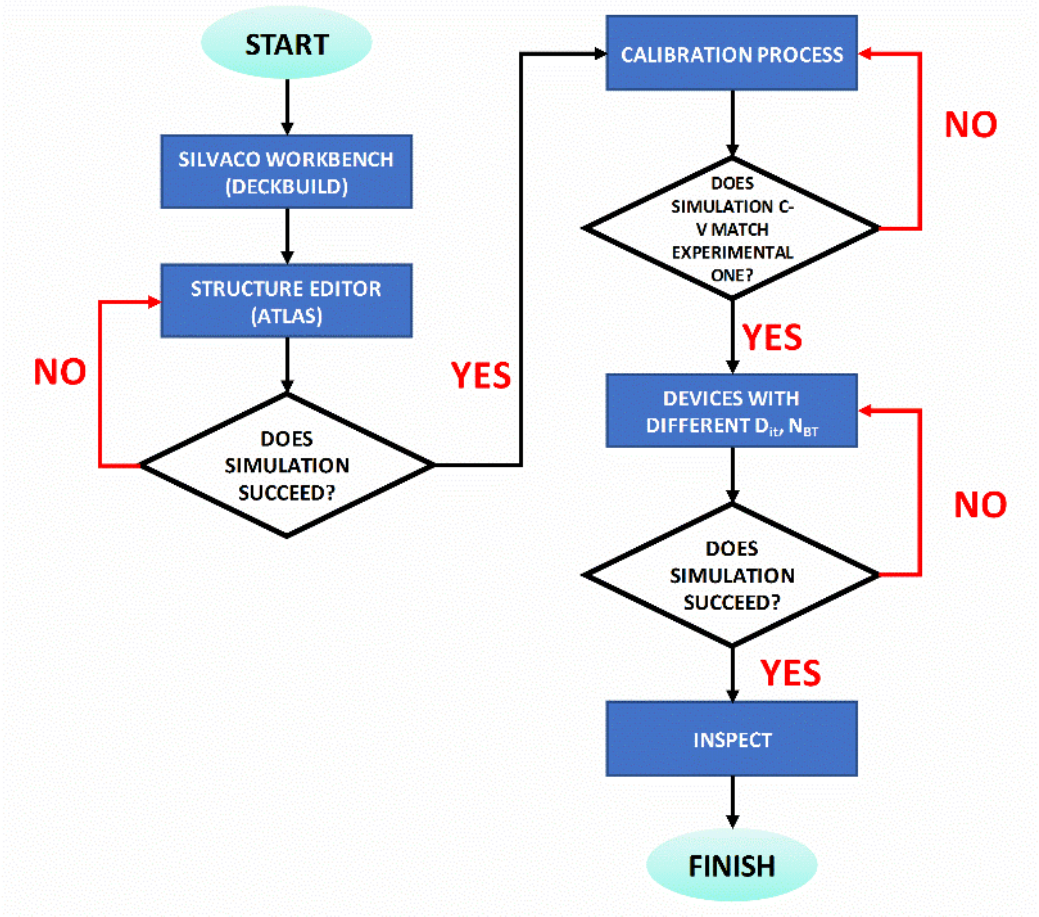

Figure S1. Flow chart of process simulation in Silvaco TCAD to study the effects of interface and border traps in metal/HfO_2/AlON/IGA structures.

Figure S1 shows the flowchart to simulate the effects of interface and border traps in metal/HfO_2/AlON/IGA MOSCAP. First, the structures of devices were constructed in Atlas using Deckbuild. To ensure all parameters used in the simulation are correct, we conducted the C-V calibration. Finally, D_{it} and N_{BT} were varied to investigate their effects on the capacitance-voltage behaviors.

B. The effects of gate metals on Metal/HfO_2/AlON/InGaAs structures.

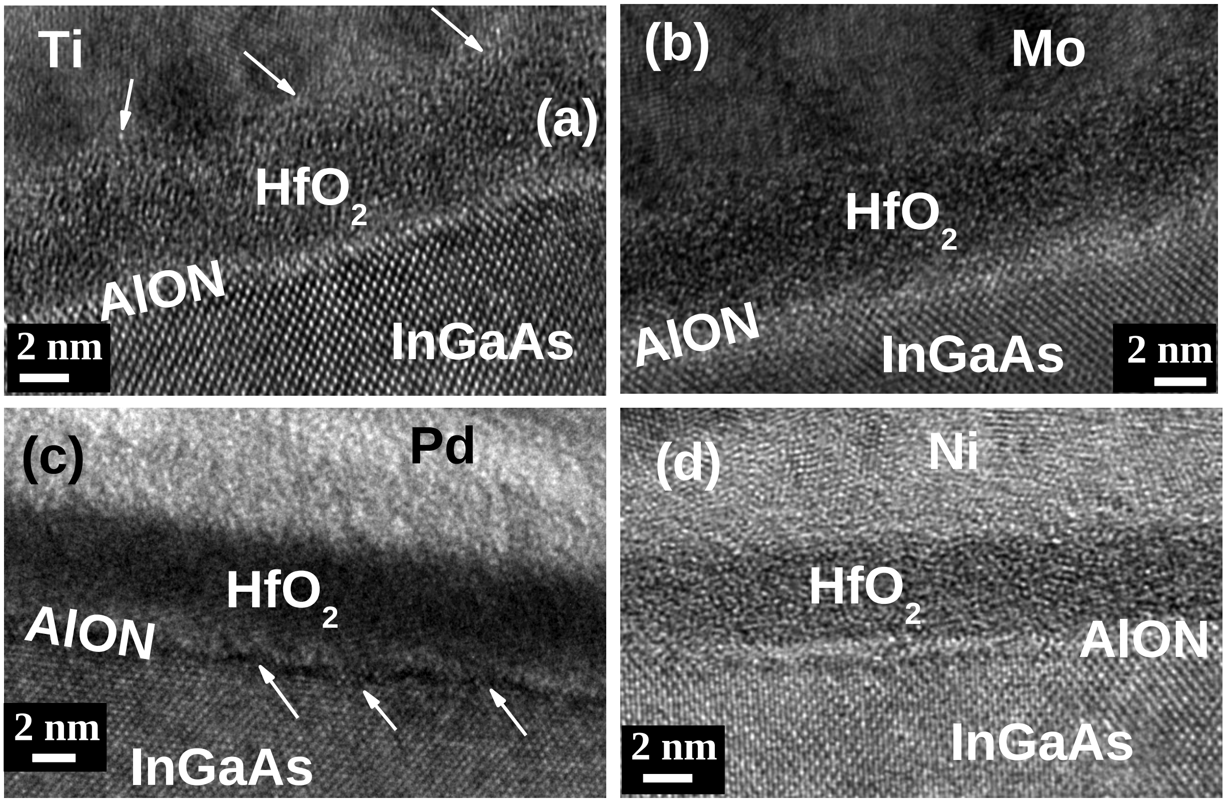

Figure S2. TEM cross sections of metal/HfO_2/AlON/IGA structures. Metal: (a) Ti, (b) Mo, (c) Pd, (d) Ni. The Mo/HfO_2 and Ni/HfO_2 interfaces were found to have no interdiffusion, while the oxidation was observed at Ti/HfO_2 interface for Ti sample (indicated by white arrows). White arrows in Figure S2(c) demonstrate that Pd gate metal induces many defects in AlON passivation layer.

Figure S2 illustrates TEM cross sections of metal/HfO_2/AlON/IGA structures. It is seen that Figure S2(a) shows the roughness at Ti/HfO_2 interface, indicating the reaction of Ti and HfO_2. This reaction facilitates the formation of border traps in HfO_2, illustrating the large frequency dispersion in Figure 2(a). The sharp interface of Mo and Ni samples in Figure S2(b) and Figure S2(d) demonstrates the stability of the Mo/HfO_2/AlON/IGA and Ni/HfO_2/AlON/IGA structures. The

TEM results are in agreement with the low D_{it} and low N_{BT} extracted in Figure 4(e) and Figure 4(f). For the Pd sample, literature reported that Pd induced the reaction of HfO_2 and IGA [1]. In our study, this phenomenon was observed in Figure S2(c), confirming in the increase of the thickness of AlON/IGA interface. The defects observed at AlON/IGA interface in Figure S2(c) specify the reasons of high D_{it} and N_{BT} shown in Figure 4(e) and 4(f), and low permitivity of the high-k layer shown in Figure 3(b).

Reference

1. Yoshida, S., et al., *Systematic study of interfacial reactions induced by metal electrodes in high-k/InGaAs gate stacks.* Applied Physics Letters, 2016. **109**(17).
